# Impact of BMI on patient outcome in acute myeloid leukaemia patients receiving intensive induction therapy: a real-world registry experience

**DOI:** 10.1038/s41416-023-02362-3

**Published:** 2023-08-04

**Authors:** Julius C. Enßle, Sebastian Wolf, Sebastian Scheich, Sarah Weber, Michael Kramer, Leo Ruhnke, Christoph Schliemann, Jan-Henrik Mikesch, Stefan Krause, Tim Sauer, Maher Hanoun, Hans Christian Reinhardt, Sabrina Kraus, Martin Kaufmann, Mathias Hänel, Lars Fransecky, Andreas Burchert, Andreas Neubauer, Martina Crysandt, Edgar Jost, Dirk Niemann, Kerstin Schäfer-Eckart, Gerhard Held, Ulrich Kaiser, Maxi Wass, Markus Schaich, Carsten Müller-Tidow, Uwe Platzbecker, Claudia D. Baldus, Martin Bornhäuser, Christoph Röllig, Hubert Serve, Study Alliance Leukemia (SAL), Björn Steffen

**Affiliations:** 1 Department of Medicine II, Hematology and Oncology, University Hospital Frankfurt, Goethe-University, Frankfurt am Main, Germany; 2 Department of Internal Medicine I, University Hospital Dresden, Dresden, Germany; 3 Department of Medicine A, University Hospital Münster, Münster, Germany; 4 Department of Hematology and Medical Oncology, University Hospital Erlangen, Erlangen, Germany; 5 Department of Medicine V, Hematology, Oncology and Rheumatology, University Hospital Heidelberg, Heidelberg, Germany; 6 Department of Hematology and Stem Cell Transplantation, University Hospital Essen, Essen, Germany; 7 Department of Internal Medicine II, University Hospital Würzburg, Würzburg, Germany; 8 Department of Hematology, Oncology and Palliative Medicine, Robert-Bosch-Hospital, Stuttgart, Germany; 9 Department of Internal Medicine III, Chemnitz Hospital, Chemnitz, Germany; 10 Department of Internal Medicine II, University Hospital Schleswig-Holstein, Kiel, Germany; 11 Department of Hematology, Oncology and Immunology, University Hospital Marburg, Marburg, Germany; 12 Department of Internal Medicine IV, University Hospital RWTH Aachen, Aachen, Germany; 13 Department of Hematology/Oncology and Palliative Medicine, Ev. Stift St. Martin, Koblenz, Germany; 14 Department of Internal Medicine 5, Hospital Nürnberg, Paracelsus Medizinische Privatuniversität, Nürnberg, Germany; 15 Department of Internal Medicine I, Westpfalz Klinik, Kaiserslautern, Germany; 16 Department of Hematology and Oncology, St. Bernward Hospital, Hildesheim, Germany; 17 Department of Internal Medicine IV, University Hospital Halle (Saale), Halle (Saale), Germany; 18 Department of Hematology, Oncology and Palliative Medicine, Rems-Murr-Kliniken, Winnenden, Germany; 19 Department for Internal Medicine I, University Hospital Leipzig, Leipzig, Germany

**Keywords:** Acute myeloid leukaemia, Epidemiology

## Abstract

**Background:**

Acute myeloid leukaemia (AML) is treated with intensive induction chemotherapy (IT) in medically fit patients. In general, obesity was identified as a risk factor for all-cause mortality, and there is an ongoing debate on its impact on outcome and optimal dosing strategy in obese AML patients.

**Methods:**

We conducted a registry study screening 7632 patients and assessed the impact of obesity in 1677 equally IT treated, newly diagnosed AML patients on the outcome (OS, EFS, CR1), comorbidities, toxicities and used dosing strategies.

**Results:**

Obese patients (BMI ≥ 30) displayed a significant inferior median OS (29.44 vs. 47.94 months, *P* = 0.015) and CR1 rate (78.7% vs. 84.3%, *P* = 0.015) without differences in median EFS (7.8 vs. 9.89 months, *P* = 0.3) compared to non-obese patients (BMI < 30). The effect was predominantly observed in older (≥60 years) patients. Obesity was identified as an independent risk factor for death, and obese patients demonstrated higher rates of cardiovascular or metabolic comorbidities. No differences for OS, EFS, CR1 or treatment-related toxicities were observed by stratification according to used dosing strategy or dose reduction.

**Conclusions:**

In conclusion, this study identifies obesity as an independent risk factor for worse OS in older AML patients undergoing curative IT most likely due to obesity-related comorbidities and not to dosing strategy.

## Introduction

Acute myeloid leukaemia (AML) is a life-threatening haematological malignancy with a median 5-year overall survival (OS) ranging from 60% in younger, medically fit patients to 23% in elderly, comorbid patients [1]. Major risk factors affecting the OS beside the patient’s age are molecular and cytogenetic aberrations [2]. Although novel targeted drugs are available for a subset of AML patients with genetic lesions, chemotherapy comprising cytarabin and daunorubicin (“3 + 7”) is the backbone of intensive therapy in eligible patients [2].

In general, cancer patient outcome and mortality are also influenced by patient-specific features—such as obesity indicated by a patient’s body mass index (BMI) ≥30 [3]. The global incidence of obesity is increasing steadily worldwide and various studies have assessed the role of obesity as a risk factor in AML patients [4–10]. There is an open debate if chemotherapy may be reduced to prevent additional toxicity or whether this would rather lead to a systemic underdosage and undertreatment of obese patients [11]. While most studies report no significant impact of obesity on OS and no significant differences in chemotherapy-induced toxicity, only some provide specific data on dosing strategies and the impact of dose reduction on outcome and toxicity in AML patients [6–8, 12, 13]. Hence, the question of dose reduction in obese AML patients remains open.

We conducted a retrospective registry study by screening real-world data of 7632 AML patients and analysing 1677 patients within the German *Study Alliance Leukaemia* (SAL)-AML registry, who were treated equally with intensive induction chemotherapy (IT) for newly diagnosed AML to assess the impact of obesity on patient outcome and the consequences of different dosing strategies as well as dose reduction.

## Materials and methods

### Study design and definitions

In this retrospective registry study, we analysed AML patients who underwent IT—with available dosage information and calculable dosing strategy—for the treatment of newly diagnosed acute myeloid leukaemia (AML) documented in the German SAL-AML registry from February 2007 to October 2019. De novo AML was defined by blast count of ≥20% in the bone marrow and/or peripheral blood or the occurrence of AML-defining genetic aberrations [2, 14]. If AML evolved from myelodysplastic syndrome or myeloproliferative neoplasia, AML was classified as secondary AML (sAML), and if AML was diagnosed after a previous chemotherapy or radiation therapy, patients were labelled as therapy-related AML (tAML). For this analysis, we selected all patients who received IT consisting of cytarabine 100 mg/m^2^ as continuous infusion for seven consecutive days as well as daunorubicin 60 mg/m^2^ on 3 days (“3 + 7” regimen). To assign patients to dosage groups, the absolute chemotherapy dosage was compared with the calculated dosage for different dosing strategies (with a +/− 5% margin to correct for rounding errors). These dosing strategies included total body weight (TBW), dosage capped at 2 m^2^ body surface area (BSA) according to DuBois/DuBois [15], idealised body weight (IBW) and adjusted idealised body weight (AIBW) (Supplementary information for detailed calculation formula) [16, 17]. Patients were excluded if their documented absolute chemotherapy for IT did not match one of the above-described dosing strategies, if they received cytarabine prephase treatment or if dosing data was missing (Supplementary Fig. S1).

Weight and height were assessed on day 1 of the treatment regimen. In accordance with the criteria of the world health organisation (WHO), normal weight was defined as BMI < 25, overweight as BMI 25–30 and obesity as BMI ≥ 30 [18]. Comorbidities were documented on the day of inclusion into the registry. Treatment-related toxicities were assessed after the first and second courses of induction therapy. Other patient characteristics depicted in Table 1 were assessed on the day of the initial diagnosis.

**Table 1:** Patient characteristics and outcome parameters.

	All patients	BMI<30	BMI≥30	*P* value
No. of patients	1677	1296	381	
Patient characteristics				
Age in years, median (range)	57 (16–85.00)	57 (18–85)	59 (16–80)	0.005
Male sex, *n* (%)	932 (55.6)	726 (56.0)	206 (54.1)	0.539
Weight in kg, median (range)	78 (41–150)	73 (41–113)	100 (71–150)	<0.001
Height in cm, median (range)	173 (138–203)	173 (138–203)	171 (147–199)	0.002
BSA in m^2^, median (range)	1.92 (1.35–2.77)	1.87 (1.35–2.46)	2.11 (1.66–2.77)	<0.001
BMI, median (range)	25.89 (15.61–52.19)	24.62 (15.61–29.97)	32.91 (30.02–52.19)	<0.001
ECOG, *n* (%)				0.179
0	484 (28.9)	393 (30.3)	91 (23.9)	
1	954 (56.9)	722 (55.7)	232 (60.9)	
2	184 (11.0)	136 (10.5)	48 (12.6)	
3	25 (1.5)	21 (1.6)	4 (1.0)	
4	9 (0.5)	7 (0.5)	2 (0.5)	
NA	21 (1.3)	17 (1.3)	4 (1.0)	
AML type, *n* (%)				0.966
De novo	1248 (74.4)	963 (74.3)	285 (74.8)	
sAML	261 (15.6)	204 (15.7)	57 (15.0)	
tAML	161 (9.6)	124 (9.6)	37 (9.7)	
NA	7 (0.4)	5 (0.4)	2 (0.5)	
Complex caryogram, *n* (%)				0.25
Yes	193 (11.5)	148 (11.4)	45 (11.8)	
No	1326 (79.1)	1034 (79.8)	292 (76.6)	
NA	158 (9.4)	114 (8.8)	44 (11.5)	
ELN category, *n* (%)				0.163
Favourable	394 (23.5)	306 (23.6)	88 (23.1)	
Intermediate	816 (48.7)	645 (49.8)	171 (44.9)	
Adverse	292 (17.4)	219 (16.9)	73 (19.2)	
NA	175 (10.4)	126 (9.7)	49 (12.9)	
WBC GPT/l, median (range)	6.77 (0.00–433.90)	7.02 (0.00–433.90)	6.00 (0.00–306.10)	0.328
HB mmol/l, median (range)	5.59 (0.39–10.10)	5.59 (0.47–10.10)	5.53 (0.39–9.75)	0.943
PLT GPT/l, median (range)	57.00 (0.01–1134.00)	57.00 (0.01–1134.00)	57.00 (0.13–631.00)	0.615
BM blasts, median (range)	60.00 (0.00–100.00)	60.00 (0.00–100.00)	59.00 (1.00–100.00)	0.379
PB blasts, median (range)	21.00 (0.00–98.00)	22.00 (0.00–98.00)	20.00 (0.00–97.00)	0.774
allo-HSCT, *n* (%)	883 (52.7)	704 (54.3)	179 (47.0)	0.014
Two cycles of IT, *n* (%)	1095 (65.3)	862 (66.5)	233 (61.2)	0.061
Outcome parameters				
CR1, *n* (%)	1392 (83.0)	1092 (84.3)	300 (78.7)	0.015
Median OS, months (range)	40.68 (0.26–103.91)	47.94 (0.26–103.91)	29.44 (0.39–89.85)	0.0017
Median EFS, months (range)	9.4 (0.39–87.38)	9.89 (0.26–93.75)	7.8 (0.39–87.38)	0.3
Patient comorbidities				
Cardiovascular, *n* (%)	548 (32.7)	337 (26.0)	211 (55.4)	<0.001
Gastrointestinal, *n* (%)	139 (8.3)	106 (8.2)	33 (8.7)	0.846
Metabolic, *n* (%)	90 (5.4)	59 (4.6)	31 (8.1)	0.009
Pulmonary, *n* (%)	129 (7.7)	104 (8.0)	25 (6.6)	0.405
Treatment-related toxicities				
Serum creatinine elevation, *n* (%)	38 (2.3)	22 (1.7)	16 (4.2)	0.007
Bleeding, *n* (%)	54 (3.2)	41 (3.2)	13 (3.4)	0.938
Serum bilirubin elevation, *n* (%)	39 (2.3)	30 (2.3)	9 (2.4)	1
Infection, *n* (%)	918 (54.9)	716 (55.4)	202 (53.2)	0.481
Cardiac toxicity, *n* (%)	49 (2.9)	34 (2.6)	15 (3.9)	0.243
ALAT/ASAT elevation, *n* (%)	51 (3.0)	45 (3.5)	6 (1.6)	0.084
Dosing strategies				
Used dosing strategies, *n* (%)				<0.001
TBW	1521 (90.7)	1223 (94.4)	298 (78.2)	
Capped 2qm	103 (6.1)	49 (3.8)	54 (14.2)	
AIBW	29 (1.7)	6 (0.5)	23 (6.0)	
IBW	24 (1.4)	18 (1.4)	6 (1.6)	

*AIBW* adjusted idealised body weight, *ALAT* alanine-aminotransferase, *allo-HSCT* allogenous hematopoietic stem cell transplantation, *AML* acute myeloid leukaemia, *ASAT* aspartate-aminotransferase, *BM* bone marrow, *BMI* body mass index, *BSA* body surface area, *CR1* first complete remission rate, *ECOG* Eastern cooperative oncology group, *EFS* event-free survival, *ELN* European leukaemia network, *HB* haemoglobin, *IBW* idealised body weight, *IT* induction treatment, *OS* overall survival, *PB* peripheral blood, *PLT* platelets, *N* number, *NA* not annotated, *sAML* secondary AML, *tAML* therapy-associated AML, *TBW* total body weight, *WBC* white blood cells.

*P* values indicate the difference between obese (BMI ≥ 30) and non-obese (BMI < 30) patients. Cardiac comorbidities comprise presence of arterial hypertonus, cardiac arrythmias or cardiac valve disease. Metabolic comorbidites combine the presence of chronic kidney injury and diabetes.

### Endpoints of the study

The primary endpoint of the study was OS defined as the time from diagnosis to death by any cause. Secondary endpoints were event-free survival (EFS) defined as the time from diagnosis to treatment failure (no complete remission after two IT cycles), relapse or death of any cause, and non-relapse-or-refractory-related mortality (NRRrM). First complete remission (CR1) was defined as complete remission (CR, <5% bone marrow (BM) blasts, absence of peripheral blood blasts and peripheral neutrophil counts >1000/µl together with platelet counts >100,000/µl) or CR with incomplete haematologic recovery (neutrophil counts <1000/µl and platelet counts <100,000/µl) after IT. Every patient declared informed written consent and standards of good clinical and scientific practice were followed at all times. As a non-interventional study documenting disease and treatment characteristics as well as outcome data, all ethics committees of the 46 participating centres in Germany approved the study protocol of the SAL-AML registry with local ethics approval and the study is registered on clinicaltrials.gov (NCT03188874). All patients declared informed consent.

### Statistical analysis

For statistical analysis, R version 4.0.3 (The R Foundation for Statistical Computing) was used [19]. Continuous variables were compared with the Mann–Whitney *U* test for two independent groups and Kruskal–Wallis test for three or more independent groups, and categorical variables with the Fisher’s exact test and the chi-square test. Cox proportional hazards for obesity status and clinically established risk factors were calculated for multivariate analysis using the *survival* package version 3.1 [20]. Survival analysis by the Kaplan–Meier method and comparison by Log-rank test were carried out with the *survival* and *survminer* package version 3.1 and 0.4.6 [20, 21]. Cumulative incidence and competing risk was analysed with the *cmprsk* package version 2.2 [22]. Cumulative incidences were compared using Gray’s test.

## Results

### Patient characteristics

According to our inclusion criteria, we identified 1677 predominantly male (*n* = 932, 55.6%) AML patients in the SAL-AML registry for further analysis (Supplementary Fig. S1). Detailed patient characteristics are depicted in Table 1. The median age was 57 years (range 16–85) and a majority of patients was assigned to Eastern Cooperative Oncology (ECOG) status of 1 (*n* = 954, 56.9%). Most patients were diagnosed with de novo AML (*n* = 1248, 74.7%) and intermediate (*n* = 816, 48.7%) cytogenetic risk according to the European Leukaemia Network (ELN) classification. At the time of diagnosis, the median white blood cell count (WBC) of all patients was 6.77 GPT/l (range 0–433.9), the median bone marrow blast count was 60% (range 0–100) and the median peripheral blood blast count was 21% (range 0–98). Approximately half (*n* = 883, 52.7%) of the patient cohort underwent allogenous stem cell transplantation (allo-HSCT) during the course of the treatment. When stratified for obese (BMI ≥ 30, *n* = 381, 22.7%) and non-obese (BMI < 30, *n* = 1296, 77.3%), no significant differences were observed for sex or ECOG status, but patients within the obese subgroup displayed a slightly higher median age compared with non-obese patients (59 vs. 57 years, *P* = 0.005). Further, both patient groups did not differ significantly with respect to type of AML, ELN risk category or WBC and blast counts. However, less obese patients received allo-HSCT as consolidation therapy compared to non-obese patients (*n* = 179, 47% vs. *n* = 704, 54.3%, *P* = 0.014).

### Outcome analysis

**Fig. 1:**
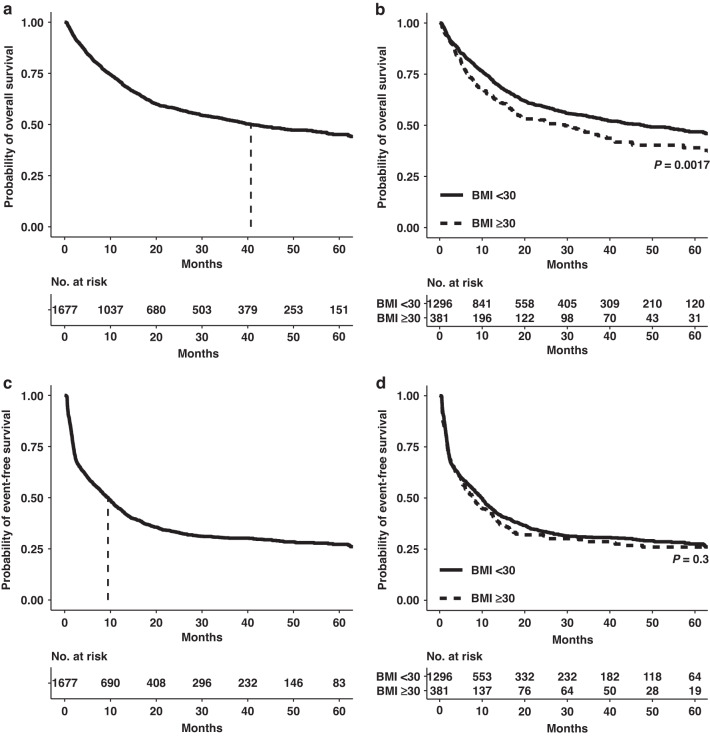
Impact of BMI on overall and event-free survival. **a** Kaplan–Meier estimates for overall survival (OS). **b** OS stratified for obese (BMI ≥ 30) and non-obese (BMI < 30) patients. **c** Kaplan–Meier estimates for event-free survival (EFS). **d** EFS stratified for obese (BMI ≥ 30) and non-obese (BMI < 30) patients.

With a median follow-up time of 34.93 months (range 0.26–103.9), the median OS of all patients was 40.68 months (range 0.26–103.9) (Fig. 1a and Table 1). Notably, obese patients had a significantly shorter overall survival compared to non-obese patients (median OS 29.44 months vs. 47.94 months, *P* = 0.0017) (Fig. 1b and Table 1) with no differences by sex (data not shown). When the non-obese patient cohort was further subdivided in normal weight (BMI < 25) and overweight but not obese (BMI 25–30), obese patients displayed a significant worse median OS compared to normal-weight patients (29.44 months vs. 58.62 months, *P* = 0.0003). Overweight patients also experienced shorter median OS compared to normal-weight patients (33.68 months vs. 58.62 months, *P* = 0.016 [global *P* = 0.0003]) (Supplementary Fig. S2A). Interestingly, no differences in OS were observed within the first 5 months of intensive therapy (Supplementary Fig. S2B). When censoring data for allo-HSCT, an inferior median OS was also detectable for obese patients when compared to non-obese patients (13.3 vs. 15.7 months, *P* = 0.031) (Supplementary Fig. S2C). To investigate the impact of obesity on achieving a response by IT as well as the durability of responses, event-free survival (EFS) was analysed [22]. The median EFS was 9.46 months (range 0.26–87.38 months), and there was no significant difference of median EFS between obese and non-obese patients (7.8 vs. 9.89 months, *P* = 0.3) (Fig. 1c, d). Concurrently, no significant differences in EFS were observed when the non-obese cohort was additionally divided into normal-weight and overweight patients (Supplementary Fig. S2D). The majority (*n* = 1392, 83%) of patients achieved a first complete remission (CR1). Notably, obese patients had a significantly lower CR1 rate compared to non-obese patients (*n* = 300, 78.7% vs *n* = 1092, 84.3%, *P* = 0.015) (Table 1). There was no significant difference in the number of induction cycles between obese and non-obese patients (Table 1). Since we hypothesised that a major reason of the observed survival differences in obese patients may be a higher comorbidity rate in older patients, we stratified the cohort based on age at first diagnosis into patients <60 years and ≥60 years. In addition, as patients ≥60 years only receive a second cycle of IT if they do not achieve remission after the first cycle according to German guideline recommendations, we also wondered if obese patients ≥60 years may have received less cycles of IT to prevent from potential toxicity despite their necessity for remission achievement in these cases. Interestingly, in patients <60 years of age, there were no significant differences in median OS, median EFS, CR1 rate or number of induction cycles between obese and non-obese patients (Supplementary Fig. S3A, B and Supplementary Table S1). Obese patients ≥60 years showed a lower rate of CR1 (*n* = 122, 68.5% vs. *n* = 415 77.1%, *P* = 0.028) and also displayed an inferior median OS (12.42 months vs. 22.15 months, *P* = 0.0013) compared to non-obese patients (Supplementary Fig. S3C, D and Supplementary Table S1) but did not differ in the number of a second IT cycle (Supplementary Table S1).

A multivariate cox regression analysis confirmed obesity as an independent risk factor for death (HR 1.27, [95% CI 1.07–1.51], *P* = 0.005) (Table 2). Other independent risk factors were adverse ELN risk category (HR 1.66, [95% CI 1.38–2.01], *P* < 0.001), age ≥60 years (HR 1.82, [95% CI 1.57–2.1], *P* < 0.001) and secondary or treatment-related AML (HR 1.31, [95% CI 1.11–1.54], *P* < 0.001).

**Table 2:** Multivariate cox regression analysis (HR for death).

Variable		HR	95% CI	*P* value
ELN Risk category	Favourable	0.63	0.50-0-78	<0.001
	Intermediate	reference		
	Adverse	1.66	1.38–2.01	<0.001
Age ≥60		1.82	1.57–2.1	<0.001
BMI ≥30		1.27	1.07–1.51	0.005
WBC (>100 Gpt/l)		1.28	0.97–1.67	0.078
sAML/tAML		1.31	1.11–1.54	0.001

*BMI* body mass index, *ELN* European leukaemia network, *sAML* secondary acute myleoid leukaemia, *tAML* therapy-associated AML, *WBC* white blood cells.

**Fig. 2:**
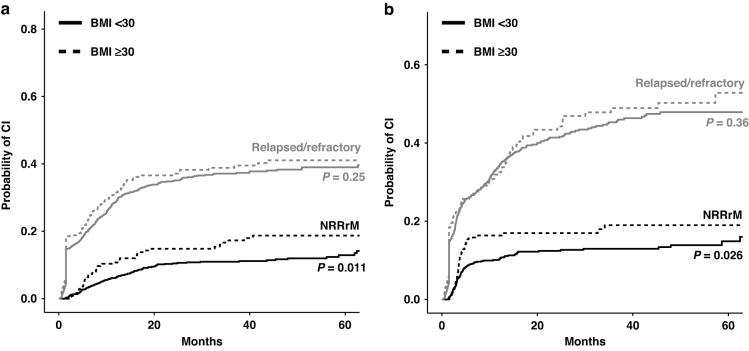
Competing risk analysis for relapsed/refractory disease and non-relapse/refractory-related mortality. **a** Competing risk analysis for cumulative incidence estimates (CIE) of relapsed or refractory diseases and non-relapse or refractory-related mortality (NRRrM). **b** Competing risk analysis for CIE of relapsed or refractory diseases and NRRrM censored for allogenous stem cell transplantation. Stratified for BMI ≥ 30 (dotted line) and BMI < 30 (solid line). *P* value indicates significance testing by Gray’s test between BMI > 30 and BMI < 30 of each CIE.

To further evaluate the inferior outcome in obese patients, we performed a competing risk analysis for cumulative incidence estimates (CIE) of relapse/refractory disease and non-relapse/refractory-related mortality (NRRrM). Interestingly, obese patients displayed a significantly higher CIE of NRRrM at 60 months (18.7% vs. 12.9%, *P* = 0.011) compared to non-obese patients, while no difference was observed for CIE of relapse/refractory disease between the two groups (Fig. 2a). Similar results were observed for patients when censoring at allo-HSCT (Fig. 2b). When this analysis was stratified by age, only patients ≥60 years showed significantly increased CIE of NRRrM without increased CIE of relapse/refractory disease. No differences in this regard were observed in younger (<60 years) patients (Supplementary Fig. S3E, F). Because we hypothesised that NRRrM is strongly influenced by comorbidities in obese patients, we investigated the comorbidities at the time of first diagnosis. Obese patients had a significantly higher incidence of cardiovascular (*n* = 211, 55.4% vs. *n* = 337, 26.0%, *P* < 0.001) and metabolic comorbidities (*n* = 31, 8.1% vs. *n* = 59, 4.6%, *P* = 0.009) (Table 1). Patients ≥60 years displayed higher rates of cardiovascular, metabolic and pulmonary comorbidities (data not shown). After the first IT cycle, obese patients displayed a higher incidence of serum creatinine elevation (*n* = 16, 4.2% vs. *n* = 22, 1.7 %, *P* = 0.007), while no differences were seen for other documented toxicities such as bleeding, serum bilirubin elevation, infection, cardiac toxicity or serum transaminase elevation (Table 1). Similar patterns were observed when patients’ comorbidities and treatment-related toxicities were analysed separately for younger and older patients (Supplementary Table S2). No differences were present between obese and non-obese patients after the second cycle of IT (Supplementary Table S3).

**Fig. 3:**
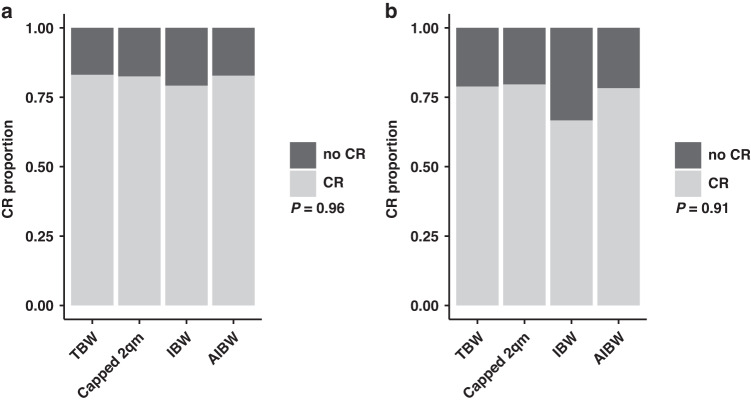
First complete response rates stratified by used dosing strategy. **a** Proportion of patients achieving first complete response (CR1) stratified by used dosing strategy. **b** Proportion of obese patients (BMI ≥ 30) achieving first complete stratified by used dosing strategy. *P* value indicates significance testing by Chi-Square Test.

Since there is an ongoing debate if chemotherapy dose should be reduced in obese patients to prevent toxicities, we stratified the patient cohort by the used dosing strategy. The majority of patients (*n* = 1521, 90.7%) received chemotherapy dosed by total body weight (TBW), while 6.14% (*n* = 103) of the patients received chemotherapy dosed by capped body surface area (BSA, capped at 2 m^2^), 1.73% (*n* = 29) were dosed by AIBW and 1.43% (*n* = 24) by IBW, respectively (Table 1). In obese patients, only 78.2% (*n* = 298) of patients received chemotherapy based on TBW, while 21.8% (*n* = 83) of the obese patients received adjusted dosing strategies (Table 1). However, no significant differences were seen for the rate of first complete remission (Fig. 3a, b) and overall survival (Supplementary Fig. S4A, B) between the different dosing strategy groups in the entire cohort as well as in the obese subgroup. No inferior survival was identified, when patients with BMI ≥ 25 were stratified regarding dose reduction <90% TBW versus >90%TBW (data not shown) [23]. Also, no significant difference in treatment toxicities was seen between the different dosing strategies (Supplementary Table S4).

## Discussion

The incidence of obesity in the global population is increasing and obesity is associated with impaired clinical outcome of oncologic patients undergoing chemotherapy [4, 24–26]. In AML, the impact of obesity on patient outcome is inconclusive and chemotherapy dosing in obese patients is a matter of debate [6–13, 23]. Therefore, we assessed the impact of obesity (BMI ≥ 30) on the outcome of AML patients undergoing induction chemotherapy for newly diagnosed AML. Obese patients had a poorer median OS and slightly lower CR1 rates when compared with non-obese patients (BMI < 30). In a multivariate analysis, BMI ≥ 30 was identified as an independent risk factor for death. Notably, there was a significantly higher CIE of NRRrM in obese patients. The obese patients also showed a higher rate of cardiovascular and metabolic comorbidities at the timepoint of treatment initiation, but the documented toxicities were not different between obese and non-obese patients. For most patients, chemotherapy was dosed by TBW while in a substantial number of obese patients, chemotherapy was reduced based on AIBW or capped BSA of 2 m^2^. However, no significant differences in the rates of CR1 or OS were observed between the various dosage groups in the entire as well as the obese patient cohort suggesting that the observed differences in CR1 and OS are not due to insufficient dosing of chemotherapy in obese patients.

In this study, we find significantly inferior OS and CR1 rates in obese AML patients. These findings are contrary to previously reported results on the impact of obesity and survival, where no significant difference was reported [6, 9, 10]. One study identified obesity as a risk factor for reduced response to IT, impaired disease-free survival and inferior OS in younger patients with de novo AML treated within prospective multicenter trials [23]. Within a subgroup analysis in patients with genetically favourable AML, obesity was shown to be associated with a reduced median OS and being also an independent risk factor for death [9]. This study also reported no difference in EFS in the whole cohort as well as in patients with ELN favourable AML. Similarly, Castillo et al. found no difference in disease-free survival between BMI groups [10]. The differences in the impact of obesity on OS between the present and previous studies may be multifactorial but could be related to specific eligibility criteria of the analysed trials. In contrast to the SAL registry, the patient population in the studies mentioned above were mostly recruited from clinical trial cohorts. Data from meta-analyses assessing potential biases show that these populations are highly selected and display a lower general risk profile when compared to real-world populations. [27]. The inclusion of low-risk profile patients with fewer comorbidities may affect the analysis on patient outcomes in the context of obesity within clinical trial cohorts.

However, the cause for the observed poorer OS of obese patients in our real-world dataset are worth discussing. Obesity in general is associated with increased mortality in cancer patients [28–31]. This association is most likely multifactorial although underlying comorbidities and host biology such as chronic inflammation, antiapoptotic effect of obesity-related hyperinsulinemia or an altered endocrine state resulting in a different intrinsic metabolic activity in obese patients may influence the course of the malignant disease [32–34]. These effects could contribute to a more aggressive tumour phenotype, impaired host defence and inferior outcome. In preclinical models, weight gain by additional fat intake enforced leukemogenesis [35]. In addition, obesity was associated with poorer OS and an increased relapse rate of patients with lymphoma undergoing high-dose chemotherapy and autologous stem cell transplantation [26]. Interestingly, we found significantly lower CR1 rates as a possible reason for lower OS in obese patients compared to non-obese patients. Such differences were not reported previously [6, 8, 9]. In contrast, previous studies reported higher CR rates in obese patients (defined as ≥130% of IBW) compared with non-obese patients as well as in patients with BMI ≥ 25 versus BMI < 25, respectively [7, 36]. To further investigate this difference in CR1 rate and OS between obese and non-obese patients, we performed a subgroup analysis in patients with <60 years and ≥60 of age. While there were no differences for OS or CR1 rate in <60 years, obese patients ≥60 years displayed a significantly lower median OS and rate of CR1.

Another possible explanation for the poorer OS and lower CR1 rate of obese patients could be an underdosing due to dose reduction in these patients. Previous studies also investigated the impact of dose modification in IT of AML patients and dose reduction is an open matter of debate [7, 11, 12, 37]. In our study, most patients received chemotherapy doses based on actual body weight, but the dose was reduced in 21.8% of obese patients. However, we observed no differences in the rate of CR1 or overall survival between the different dosage groups in the entire patient population as well as in obese patients. This is consistent with previous reports that dose reduction within the described limits does not impair response to induction chemotherapy in AML. Crysandt et al. outline a reduced OS in AML patients with BMI ≥ 25 that receive dose reduction <90% of TBW [23]. Despite that both studies (Crysandt et al. and the present) comprise of patients from the SAL-AML group (with only patients from clinical trials enrolled in the Crysandt et al. dataset), there is no overlap on the patient cohorts, and such findings were not present in our study. Given the retrospective setting of this study, there was no detailed analysis of minimal residual disease (MRD) applicable during the follow-up period. To further investigate differences in the sustainability of responses between obese and non-obese patients, such MRD analysis should be addressed in prospective studies.

There is also no evidence for increased chemotoxicity in obese patients [6–9, 36]. When our patient cohort was stratified for obesity and non-obesity, no clinically relevant significant differences for toxicity after the first and second cycle of induction chemotherapy were present. Also, there was no difference in the early mortality within the first 5 months after diagnosis that may relate to toxicity-associated adverse events. Further, analysis of the toxicity profile between older and younger AML patients stratified by obesity status did not show any major differences regarding documented treatment side effects.

Obesity is a major risk factor for and significantly associated with various medical comorbidities resulting in a generally poorer overall health status and increased all-cause mortality in the general population and particularly cancer patients [28–31, 38, 39]. Therefore, we analysed the rate of comorbidities in both obese and non-obese patients at initial diagnosis. Obese AML patients in our study population had a significantly higher rate of cardiovascular and metabolic comorbidities compared to non-obese patients. In addition, the rate of comorbidities was pronounced in older AML patients. This stands in line with previous evidence that obesity is generally—and also in cancer patients—associated with cardiovascular or diabetic medical conditions [38, 40]. When we further analysed the CIE of NRRrM in our study population, obese patients displayed a significantly higher CIE of NRRrM in contrast to non-obese patients. A separate analysis of these aspects in older and younger AML patients revealed a significant difference for the CIE of NRRrM only in patients ≥60 years. Thus, we hypothesise that obesity-associated comorbidities leading to an increased NRRrM might be one of the major drivers for poorer outcome in older obese AML patients.

However, this study also has certain limitations. Due to the retrospective nature of this study, causal interference cannot be made. In addition, stratification in both BMI groups together with further factors resulted in limited sample sizes per group. Further, there was no pharmacokinetic and -dynamic data present for these patients and the pharmacologic effects of AML IT in obese individuals are poorly understood. Also, this analysis focused on cytarabine and daunorubicin for IT agents. Novel agents, such as tyrosine-kinase inhibitors, antibody–cytotoxic drug conjugates or small-molecule inhibitors, that have increasing relevance for IT in AML, have not been assessed. We further only included patients with present and plausible dosage data and thereby also are subject to a certain selection bias. Unfortunately, no data on causes of death were available to further support our hypothesis. The body composition can be in-detail measured by radiomics metrics. Hence, further prospective studies with attending pharmacokinetic and -dynamic analyses, use of radiomics-based obesity assessment, per-protocol dosage practices and clinical documentation (including toxicity-associated long-term comorbidities) may provide a detailed insight into the cause of the observed poorer OS in obese AML patients.

In conclusion, our analysis on the largest real-world cohort demonstrates a poorer OS in older and obese patients undergoing IT for newly diagnosed AML. Based on our data, higher rates of intrinsic obesity-related comorbidities and not the dosing strategy in older obese patients may contribute to these differences. Further studies are necessary to fully elucidate the negative impact of obesity on overall survival in these patients.

### Supplementary information

Supplementary Information

## Data Availability

Data availability requests should be addressed to the corresponding author.
