# Supplementary material for: Impact of BMI on patient outcome in acute myeloid leukaemia patients receiving intensive induction therapy: a real-world registry experience
Source: Br J Cancer. 2023 Aug 4;129(7):1126–33. doi: 10.1038/s41416-023-02362-3 (PMC10539505; doi:10.1038/s41416-023-02362-3)
Supplement: Supplementary file 1 — Supplementary Information [file 41416_2023_2362_MOESM1_ESM.pdf]

Supplementary Information

Impact of BMI on patient outcome in acute myeloid leukemia patients receiving intensive induction therapy: a real-world registry experience

Julius C. Enßle, Sebastian Wolf, Sebastian Scheich, Sarah Weber, Michael Kramer, Leo Ruhnke, Christoph Schliemann, Jan-Henrik Mikesch, Stefan Krause, Tim Sauer, Maher Hanoun, Hans Christian Reinhardt, Sabrina Kraus, Martin Kaufmann, Mathias Hänel, Lars Fransecky, Andreas Burchert, Andreas Neubauer, Martina Crysandt, Edgar Jost, Dirk Niemann, Kerstin Schäfer-Eckart, Gerhard Held, Ulrich Kaiser, Maxi Wass, Markus Schaich, Carsten Müller-Tidow, Uwe Platzbecker, Claudia D. Baldus, Martin Bornhäuser, Christoph Röllig, Hubert Serve, Björn Steffen for the Study Alliance Leukemia (SAL)

Dosage calculation formulas
Supplementary figures
Supplementary figure legends
Supplementary tables (as separate .xlsx)

Dosage calculation formulas

1. Calculation of body surface are according to DuBois/DuBois:

   $BSA = 0.007184 * height(cm)^{0.725} * weight (kg)^{0.0425}$

2. Calculation of idealised body weight according to Devine:

   IBW(men) = 50kg + (0.9 * (height(cm) - 152))

   IBW(women) = 45.5kg + (0.9 * (height(cm) - 152))

3. Calculation of adjusted idealised body weight:

   AIBW = IBW + (0.4* (weight(kg) – IBW))

Supplementary Figures

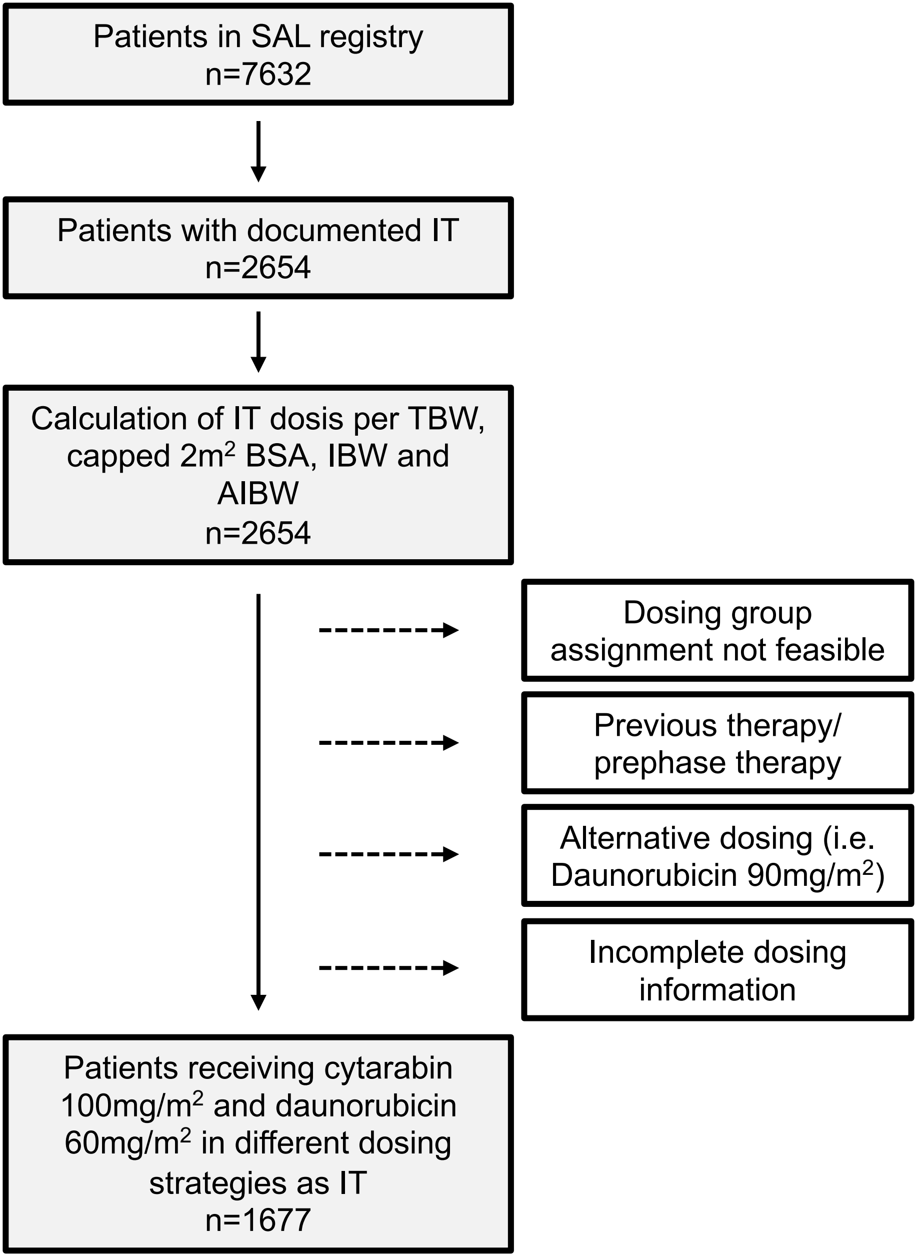
Patients in SAL registry
n=7632
Patients with documented IT
n=2654
Calculation of IT dosis per TBW, capped 2m^2 BSA, IBW and AIBW
n=2654
Dosing group assignment not feasible
Previous therapy/ prephase therapy
Alternative dosing (i.e. Daunorubicin 90mg/m^2)
Incomplete dosing information
Patients receiving cytarabin 100mg/m^2 and daunorubicin 60mg/m^2 in different dosing strategies as IT
n=1677

Figure S1

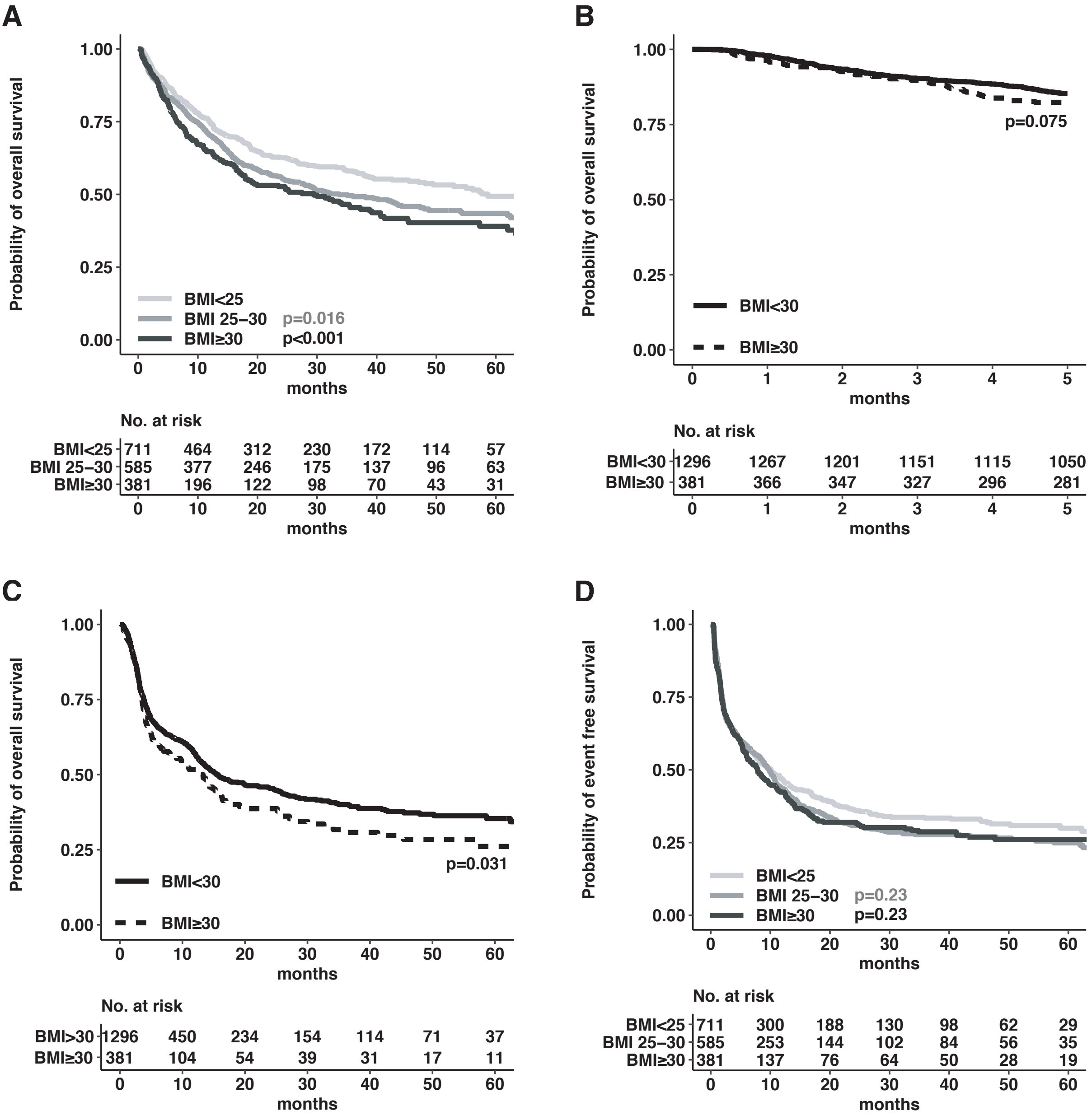
A
Probability of overall survival
1.00
0.75
0.50
0.25
0.00
BMI<25
BMI 25–30
BMI≥30
p=0.016
p<0.001
0
10
20
30
40
50
60
months
No. at risk
BMI<25 711 464 312 230 172 114 57
BMI 25–30 585 377 246 175 137 96 63
BMI≥30 381 196 122 98 70 43 31
B
Probability of overall survival
p=0.075
BMI<30
BMI≥30
0
1
2
3
4
5
months
No. at risk
BMI<30 1296 1267 1201 1151 1115 1050
BMI≥30 381 366 347 327 296 281
C
Probability of overall survival
p=0.031
BMI<30
BMI≥30
months
No. at risk
BMI>30 1296 450 234 154 114 71 37
BMI≥30 381 104 54 39 31 17 11
D
Probability of event free survival
BMI<25
BMI 25–30
BMI≥30
p=0.23
p=0.23
months
No. at risk
BMI<25 711 300 188 130 98 62 29
BMI 25–30 585 253 144 102 84 56 35
BMI≥30 381 137 76 64 50 28 19

Figure S2

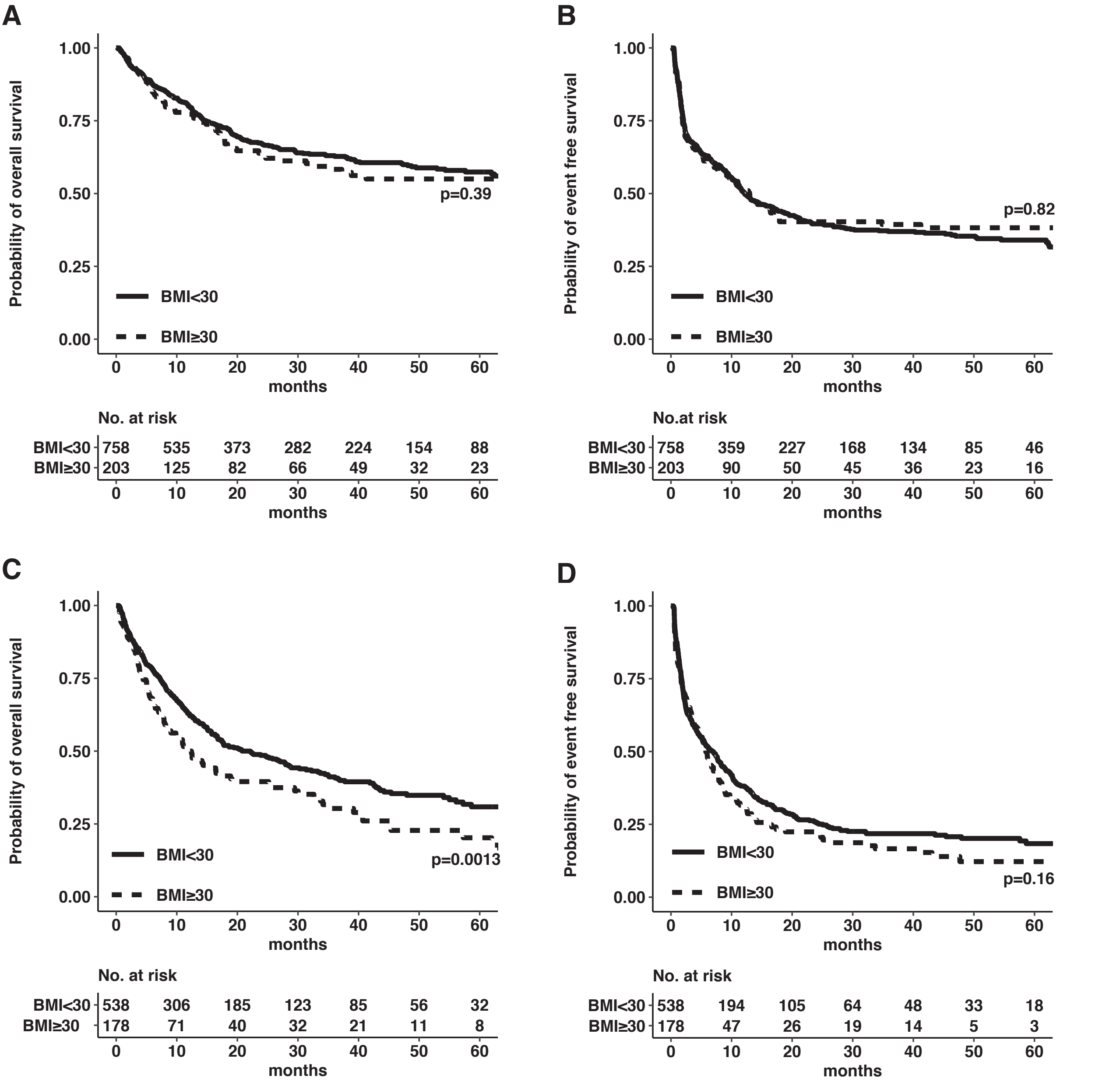

A
Probability of overall survival
1.00
0.75
0.50
0.25
0.00
0 10 20 30 40 50 60
months
p=0.39
BMI<30
BMI≥30
No. at risk
BMI<30 758 535 373 282 224 154 88
BMI≥30 203 125 82 66 49 32 23
0 10 20 30 40 50 60
months
B
Prbability of event free survival
1.00
0.75
0.50
0.25
0.00
0 10 20 30 40 50 60
months
p=0.82
BMI<30
BMI≥30
No.at risk
BMI<30 758 359 227 168 134 85 46
BMI≥30 203 90 50 45 36 23 16
0 10 20 30 40 50 60
months
C
Probability of overall survival
1.00
0.75
0.50
0.25
0.00
0 10 20 30 40 50 60
months
p=0.0013
BMI<30
BMI≥30
No. at risk
BMI<30 538 306 185 123 85 56 32
BMI≥30 178 71 40 32 21 11 8
0 10 20 30 40 50 60
months
D
Probability of event free survival
1.00
0.75
0.50
0.25
0.00
0 10 20 30 40 50 60
months
p=0.16
BMI<30
BMI≥30
No. at risk
BMI<30 538 194 105 64 48 33 18
BMI≥30 178 47 26 19 14 5 3
0 10 20 30 40 50 60
months

Figure S3

E

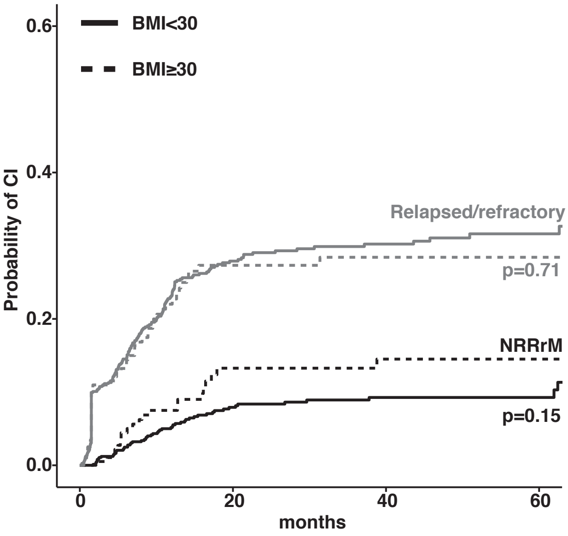

F

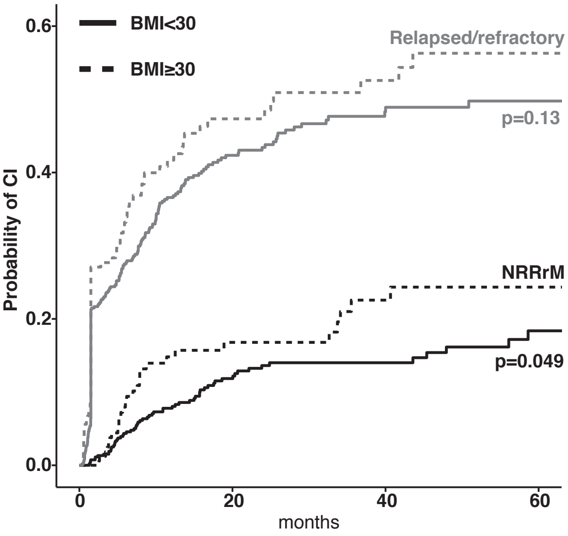

Figure S3 (continued)

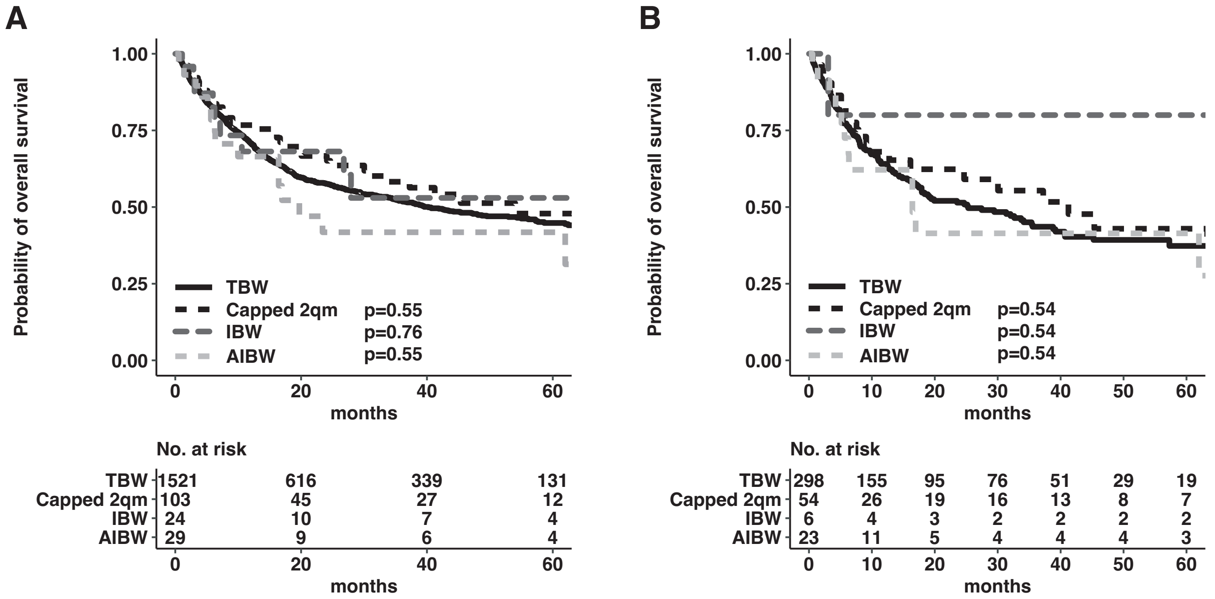
A
Probability of overall survival
1.00
0.75
0.50
0.25
0.00
TBW
Capped 2qm p=0.55
IBW p=0.76
AIBW p=0.55
0 20 40 60
months
No. at risk
TBW 1521 616 339 131
Capped 2qm 103 45 27 12
IBW 24 10 7 4
AIBW 29 9 6 4
0 20 40 60
months
B
Probability of overall survival
1.00
0.75
0.50
0.25
0.00
TBW
Capped 2qm p=0.54
IBW p=0.54
AIBW p=0.54
0 10 20 30 40 50 60
months
No. at risk
TBW 298 155 95 76 51 29 19
Capped 2qm 54 26 19 16 13 8 7
IBW 6 4 3 2 2 2 2
AIBW 23 11 5 4 4 4 3
0 10 20 30 40 50 60
months

Figure S4

Supplementary Figure Legends

Supplementary Figure 1. Schematic overview of screening and identification of relevant patients for the present study population. AIBW, adjusted-idealised body weight; BSA, body surface area; IBW, idealised body weight; IT, induction therapy; TBW, total body weight.

Supplementary Figure 2. A) Kaplan-Meier estimates for overall survival (OS) stratified by BMI<25, BMI 25-30 and BMI≥30, p- value indicates differences of each group in comparison to BMI 25-30, global LogRank p = 0.0003. B) Kaplan-Meier estimates for overall survival (OS) within the first 5 months after initial diagnosis stratified by BMI≥30 and BMI<30. C) Kaplan-Meier estimates for overall survival (OS) censored for allogenous stem cell transplantation (allo-HSCT) and stratified for obese (BMI ≥30) and non-obese (BMI<30) patients. D) Kaplan-Meier estimates for event-free survival (EFS) stratified by BMI<25, BMI 25-30 and BMI≥30, global LogRank p = 0.21. P-value indicates differences of each group in comparison to BMI 25-30.

Supplementary Figure 3. A) Kaplan-Meier estimates for overall survival (OS) stratified for obese (BMI ≥30) and non-obese (BMI<30) patients <60 years. B) Kaplan-Meier estimates for event-free survival stratified for obese (BMI ≥30) and non-obese (BMI<30) patients <60 years. C) Kaplan-Meier estimates for overall survival (OS) stratified for obese (BMI ≥30) and non-obese (BMI<30) patients ≥60 years. D) Kaplan-Meier estimates for event-free survival stratified for obese (BMI ≥30) and non-obese (BMI<30) patients ≥60 years. E) Competing risk analysis for cumulative incidence estimates (CIE) of relapsed or refractory diseases and non-relapse or refractory related mortality (NRRrM) for patients <60 years. F) Competing risk analysis for CIE of relapsed or refractory diseases and NRRrM for patients ≥60 years.

Supplementary Figure 4. A) Kaplan-Meier estimates for overall survival stratified by used dosing strategy with either total body weight (TBW), capped at 2 m^2 body surface area (BSA), idealized body weight (IBW) or adjusted-idealized body weight AIBW. B) Kaplan-Meier estimates for overall survival stratified by used dosing strategy with

either TBW, capped at 2 m^2 BSA, IBW or AIBW. P-value indicates differences of each group in comparison to TBW.

Table S1. Subgroup analysis stratified by age

	all patients	BMI<30	BMI≥30	p-value
N. patients with age < 60	961	758	203	
CR1, n (%)	855 (89.0)	677 (89.3)	178 (87.7)	0.595
Median OS, months (range)	82.78 (0.26-103.91)	82.78 (0.26-103.91)	Not reached	0.38
Median EFS, months (range)	12.38 (0.26-93.76)	12.19 (0.26-93.76)	12.98 (0.49-87.38)	0.82
Two cycles of IT, n (%)	730 (76.0)	580 (76.5)	150 (73.9)	0.493
Patients with age ≥ 60	716	538	178	
CR1, n (%)	537 (75.0)	415 (77.1)	122 (68.5)	0.028
Median OS, months (range)	17.71 (0.36-96.28)	22.15 (0.36-96.28)	12.42 (0.39-84.26)	0.0013
Median EFS, months (range)	6.27 (0.32-92.70)	6.84 (0.32-92.70)	5.75 (0.39-74.53)	0.16
Two cycles of IT, n (%)	365 (51.0)	282 (52.4)	83 (46.6)	0.21

P-values indicate differences between obese (BMI≥30) and non-obese (BMI<30) patients. BMI, Body mass index; CR1, first complete remission; EFS, Event-free survival; IT, Induction treatment; N, number; OS, Overall survival

Table S2. Patient comorbidities and treatment-related toxicities stratified by age group

	all patients	BMI<30	BMI≥30	p-value
Patient comorbidities				
N with age <60	961	758	203	
cardiovascular, n (%)	186 (19.4)	99 (13.1)	87 (42.9)	<0.001
gastrointestinal, n (%)	68 (7.1)	55 (7.3)	13 (6.4)	0.79
metabolic, n (%)	30 (3.1)	21 (2.8)	9 (4.4)	0.326
pulmonary, n (%)	54 (5.6)	46 (6.1)	8 (3.9)	0.319
n with age ≥60	716	538	178	
cardiovascular, n (%)	362 (50.6)	238 (44.2)	124 (69.7)	<0.001
gastrointestinal, n (%)	71 (9.9)	51 (9.5)	20 (11.2)	0.593
metabolic, n (%)	60 (8.4)	38 (7.1)	22 (12.4)	0.04
pulmonary, n (%)	75 (10.5)	58 (10.8)	17 (9.6)	0.746
Treatment-related toxicities after 1st IT cycle				
N with age <60	961	758	203	
serum creatinin elevation, n (%)	11 (1.1)	6 (0.8)	5 (2.5)	0.105
bleeding, n (%)	27 (2.8)	20 (2.6)	7 (3.5)	0.699
serum bilirubin elevation, n (%)	19 (2.0)	14 (1.9)	5 (2.5)	0.779
infection, n (%)	518 (54.1)	416 (55.0)	102 (50.5)	0.285
cardiac toxicity, n (%)	21 (2.2)	15 (2.0)	6 (3.0)	0.562
ALAT/ASAT elevation, n (%)	28 (2.9)	27 (3.6)	1 (0.5)	0.038
n with age ≥60	716	538	178	
serum creatinin elevation, n (%)	27 (3.8)	16 (3.0)	11 (6.2)	0.086
bleeding, n (%)	27 (3.8)	21 (3.9)	6 (3.4)	0.92
serum bilirubin elevation, n (%)	20 (2.8)	16 (3.0)	4 (2.2)	0.802
infection, n (%)	400 (55.9)	300 (55.9)	100 (56.2)	1
cardiac toxicity, n (%)	28 (3.9)	19 (3.5)	9 (5.1)	0.495
ALAT/ASAT elevation, n (%)	23 (3.2)	18 (3.4)	5 (2.8)	0.912

P-values indicate differences between obese (BMI≥30) and non-obese (BMI<30) patients. ALAT, Alanine-aminotransferase; ASAT, Aspartate-aminotransferase; BMI, Body mass index; N, number

Table S3. Treatment-related toxicities after 2nd IT cycle

	all patients	BMI<30	BMI≥30	p-value
N. patients	1089	857	232	
serum creatinin elevation, n (%)	22 (2.0)	15 (1.8)	7 (3.0)	0.335
bleeding, n (%)	24 (2.2)	20 (2.3)	4 (1.7)	0.764
serum bilirubin elevation, n (%)	34 (3.1)	23 (2.7)	11 (4.8)	0.162
infection, n (%)	578 (53.1)	448 (52.3)	130 (56.3)	0.314
cardiac toxicity, n (%)	18 (1.7)	12 (1.4)	6 (2.6)	0.329
ALAT/ASAT elevation, n (%)	45 (4.1)	36 (4.2)	9 (3.9)	0.984

P-values indicate differences between obese (BMI≥30) and non-obese (BMI<30) patients. ALAT, Alanine-aminotransferase; ASAT, Aspartate-aminotransferase; BMI, Body mass index; N, number

Table S4. Treatment-related toxicites after IT cycles stratified by used dosing strategy

	all patients	TBW	Capped 2qm	IBW	AIBW	p-value
First IT	1677	1521	103	24	29	
serum creatinin elevation, n (%)	38 (2.3)	29 (1.9)	6 (5.8)	1 (4.2)	2 (6.9)	0.019
bleeding, n (%)	54 (3.2)	51 (3.4)	3 (2.9)	0 (0.0)	0 (0.0)	0.596
serum bilirubin elevation, n (%)	39 (2.3)	33 (2.2)	4 (3.9)	2 (8.3)	0 (0.0)	0.125
infection, n (%)	918 (54.9)	840 (55.4)	50 (48.5)	16 (66.7)	12 (41.4)	0.151
cardiac toxicity, n (%)	49 (2.9)	41 (2.7)	5 (4.9)	2 (8.3)	1 (3.4)	0.25
ALAT/ASAT elevation, n (%)	51 (3.0)	49 (3.2)	2 (1.9)	0 (0.0)	0 (0.0)	0.52
Second IT	1089	987	79	10	13	
serum creatinin elevation, n (%)	22 (2.0)	20 (2.0)	2 (2.5)	0 (0.0)	0 (0.0)	0.901
bleeding, n (%)	24 (2.2)	23 (2.3)	0 (0.0)	0 (0.0)	1 (7.7)	0.273
serum bilirubin elevation, n (%)	34 (3.1)	29 (2.9)	4 (5.1)	0 (0.0)	1 (7.7)	0.511
infection, n (%)	578 (53.1)	526 (53.3)	40 (50.6)	6 (60.0)	6 (46.2)	0.883
cardiac toxicity, n (%)	18 (1.7)	16 (1.6)	2 (2.5)	0 (0.0)	0 (0.0)	0.857
ALAT/ASAT elevation, n (%)	45 (4.1)	38 (3.9)	4 (5.1)	2 (20.0)	1 (7.7)	0.068

P-values indicate differences between dosage groups. AIBW, Adjusted idealised body weight; ALAT, Alanine-aminotransferase; ASAT, Aspartate-aminotransferase; BMI, Body mass index; IBW, idealised body weight; IT, Induction treatment; TBW, total body weight
